# Dose escalation using ultra-high dose IMRT in intermediate risk prostate cancer without androgen deprivation therapy: preliminary results of toxicity and biochemical control

**DOI:** 10.1186/1756-9966-32-103

**Published:** 2013-12-13

**Authors:** Maria Grazia Petrongari, Valeria Landoni, Biancamaria Saracino, Sara Gomellini, Stefano Arcangeli, Giuseppe Iaccarino, Paola Pinnarò, Giorgio Arcangeli, Lidia Strigari

**Affiliations:** 1 Department of Radiation Oncology, Regina Elena National Cancer Institute, Rome, Italy; 2 Laboratory of Medical Physics and Expert Systems, Regina Elena National Cancer Institute, Rome, Italy

**Keywords:** Prostate cancer, Intermediate risk, Dose escalation, Absence of ADT, Toxicity, Outcome

## Abstract

**Background:**

To investigate the feasibility of dose escalation (86 Gy at 2 Gy/fraction) with intensity modulated radiation therapy (IMRT) in intermediate-risk prostate cancer without androgen deprivation therapy.

**Methods:**

Patients with histologically proven adenocarcinoma of the prostate, intermediate prognostic category, were enrolled in this study. Early and late toxicity were scored according to the Cancer Therapy Evaluation Program, Common Terminology Criteria for Adverse Events, Version 3.0. Treatment outcome was stated in terms of biochemical failure, biopsy result and clinical failure.

**Results:**

39 patients with a median follow-up of 71 months were analyzed. No patient experienced G3 or G4 acute gastrointestinal (GI) or genitourinary (GU) toxicity. G2 acute GI and GU toxicity were observed in 17 (44%) and 20 (51%) patients, respectively. Fourteen patients (36%) did not experience acute GI toxicity and 4 patients (10%) did not experience acute GU toxicity. G2 late GI bleeding occurred in 7 of 39 patients (18%). Both G3 and G4 late GI toxicity were seen only in one patient (2.5%). Two patients (5%) experienced G2 late GU toxicity, while G3 late GU toxicity occurred in 3 patients (8%). The 5-year actuarial freedom from biochemical failure (FFBF) was 87%. Thirty-four patients (87%) did not show biochemical relapse. Seventeen patients (44%) underwent biopsy two year after radiotherapy; of these only two were non-negative and both did not show evidence of biochemical disease.

**Conclusions:**

IMRT treatment of patients with localized intermediate-risk prostate cancer at high dose levels without using androgen deprivation therapy (ADT) seems to give good disease control. Nevertheless, future trials should aim at further decreasing toxicity by exploiting image guidance techniques and by reducing the dose delivered at the interface between organs at risk and prostate.

## Introduction

The use of dose escalation in radiation therapy, with doses ranging from 74 to 80 Gy, has shown an improvement in the outcome of prostate cancer when compared with conventional doses, as reported in large retrospective studies [1,2] and in some prospective randomized trials [3-8]. Moreover, the dose–response curve of prostate cancer showed an optimal disease control in the region of ultra-high dose levels (≥ 80 Gy) [9]. As a matter of fact, dose escalation has improved distant metastasis-free survival (DMFS) and cancer-specific survival (CSS) [10-13]. However, the use of three-dimensional conformal radiation therapy (3D-CRT) for dose escalation is limited by side effects [3-7,14]; while intensity-modulated radiation therapy (IMRT) generally decreases treatment-related morbidity by producing steeper dose-gradients [13,15-17]. At MSKCC [17,18] the feasibility of dose escalation from 81 Gy to 86.4 Gy at 1.8 Gy/fraction in localized prostate cancer in association with short course Androgen Deprivation Therapy (ADT) has been investigated, suggesting that ultra-high dose regimen is well tolerated and reporting an excellent biochemical control. However the role and the optimal duration of ADT with dose escalated radiation therapy still remains controversial.

The aim of our paper is to report the outcome of a dose-escalation study with an ultra-high dose of 86 Gy at 2 Gy/fraction with IMRT technique in intermediate-risk prostate cancer patients, without the use of ADT, in terms of toxicity and biochemical control.

## Methods

This is a single institution prospective phase II study approved by Regina Elena National Cancer Institute, Ethical Committee. Patients enrolled in the study belonged to the intermediate prognostic category according to the National Comprehensive Cancer Network classification system (http://www.nccn.com) which included patients with stage T2b-T2c tumors, and PSA >10 ng/ml but ≤ 20 ng/ml, and Gleason score 7. The clinical characteristics of patients and tumors are shown in Table 1.

**Table 1:** Clinical characteristics of patients and tumor staging

		
Age (years)		
	Median (range)	72 (53–77)
Follow-up (mos)		
	Median (range)	71 (32.8-93.6)
Stage (N /%)		
	T1c	1 (2.5%)
	T2a	11 (28%)
	T2b	15 (38.5%)
	T2c	12 (31%)
Gleason score		
	<=6	13 (33.3%)
	7 (3 + 4)	20 (51.3%)
	7 (4 + 3)	6 (15.4%)
% Biopsy core		
	0-24%	12 (31%)
	25-49%	16 (41%)
	50-74%	10 (26%)
	75-100%	1 (2%)
iPSA		
	<10	37 (95%)
	10–19.9	2 (5%)

Inclusion criteria were: 1) age <80 years; 2) histological proof of prostate adenocarcinoma at intermediate risk; 3) risk of lymph node involvement < 15%, according to Roach formula, or absence of adenopathy assessed by CT and/or MRI; 4) WHO performance status < 2; 5) no previous pelvic radiotherapy; 6) no previous prostate surgery; 7) no previous hormonal therapy; 8) no previous malignant tumors, with the exception of adequately treated cutaneous carcinomas; 9) declared availability to comply with the planned follow-up examinations; 10) written informed consent. All patients were free of ADT treatment. Written informed consent was signed by all patients. Patients underwent a CT simulation in the prone position by using a customized device for immobilization. A CT scan was performed at 5 mm intervals from L4/L5 to 5 cm below the ischial tuberosities. Patients were asked to void the rectum before simulation and before each treatment session, also with the use of glycerine suppositories or enemas. The bladder had to be taken at middle filling by voiding it 1.5 hours before simulation and daily before each treatment session. The acquired images were then transferred to the Eclipse (v.8.9) treatment planning system. The clinical target volume (CTV) consisted of the prostate and entire seminal vesicles, the planning target volume (PTV) was obtained by adding 1 cm margin in all directions except toward the rectum, where the margin was reduced to 0.6 cm according to our institutional policy [19]. The rectal and bladder walls were contoured as critical normal structures, in particular, the rectum was outlined from the sigmoid flexure to the anal margin. Patients were treated with a 15 MV five-field sliding window IMRT technique. The beam arrangement was: posterior (0°), right posterior oblique (75°), right anterior oblique (135°), left anterior oblique (225°) and left posterior oblique (285°). Plans were optimized to give at least 95% and 90% of the prescribed dose to CTV and PTV, respectively. The maximum dose heterogeneity within the PTV was set at 17% (from 90% to 107%). No constraints were applied to the overlapping volume between the PTV and rectum, which was treated as PTV. Dose-volume constraints were set for rectal and bladder walls and femoral heads. Dose-volume constraints were: maximum 70 Gy, 50 Gy and 40 Gy to 30%, 50% and 60% of the rectal wall volume, respectively, maximum 70 Gy and 50 Gy to 50% and 70% of the bladder wall volume, respectively, and maximum 55 Gy to 70% of the femoral heads. The normal tissue planning limits were based on our prior experience and on previously published studies [20-25]. Dose-volume histograms were recorded for all patients. Patients were treated with Varian 2100 linear accelerators (Varian Associates, Palo Alto, CA) equipped with 120-leaf multi-leaf collimators. The accuracy of the set-up was monitored daily by verifying the position of the isocenter comparing skeletal landmarks on orthogonal portal images acquired with an electronic portal imaging device (EPID) to the digitally reconstructed radiography (DRRs).

### Study endpoints

The primary endpoint of our study was gastrointestinal (GI) and genitourinary (GU) toxicity. Early and late toxicity data were scored according to the Cancer Therapy Evaluation Program, Common Terminology Criteria for Adverse Events, Version 3.0 [26]. Grade 1–4: Grade 1 (mild) - asymptomatic or mild symptoms requiring only clinical or diagnostic observation; Grade 2 (moderate) - minimal, local or noninvasive intervention indicated; Grade 3 (severe) - severe or medically significant but not immediately life-threatening requiring hospitalization, prolonging hospitalization or affecting activities of daily living; Grade 4- life-threatening consequences requiring urgent intervention. Acute side effects occurred during the course of radiation or within 90 days of its completion. Late toxicity was defined as rectal or urinary symptoms occurring or persisting 6 months or more after completing radiotherapy. The secondary endpoints were biochemical failure, biopsy result and clinical failure. The freedom from biochemical failure (FFBF) was defined as the time interval from the first day of radiotherapy to the biochemical relapse, the scores are according to the most recent Phoenix definition of nadir PSA +2 ng/ml [27]. The histological diagnosis of the prostate biopsy at 2-years post-radiotherapy was classified as positive (prostatic adenocarcinoma without typical radiation-induced changes), negative (no evidence of carcinoma) or indeterminate (severe treatment effects).

### Baseline and follow-up

All patients were prostate adenocarcinoma pre-treatment biopsy proven. Baseline staging was assessed by initial PSA (iPSA) levels, digital rectal examination (DRE), transrectal ultrasound images, abdomino-pelvic CT, chest RX/CT and bone scan. At baseline, patients were asked to answer questions about their urinary symptoms according to the International Prostate Symptoms Score (IPSS) questionnaire [28]. Patients were monitored weekly during the course of radiotherapy, after 2 and 6 months from the end of the treatment, and then every six months until the second year of follow-up. Afterwards patients were monitored annually. PSA evaluation and DRE were performed at each follow-up visit and a report was drafted, with special emphasis on treatment-related morbidity, which recorded the worst toxicity score for each patient. In case of an increased PSA and/or suspected clinical local relapse (new or increasing palpable prostate nodule) or distant failure (bone pain, low extremity edema, unjustified dyspnea, etc.), the usual diagnostic imaging procedures or prostate biopsies were carried out. All patients underwent a sextant prostate re-biopsy after at least 2 years after the radiation treatment.

### Statistical analysis

For all measured endpoints, patients were censored at the time of the specific event. Actuarial curves of the length of time until late toxicity or biochemical failure were calculated by the Kaplan-Meier product-limit method. All times were calculated from the first day of radiotherapy. Differences between dosimetric parameters between groups were evaluated by a Mann–Whitney test.

## Results

### Patients and dosimetry

From January 2005 to April 2010 39 patients with histologically proven adenocarcinoma of the prostate were enrolled in an IMRT dose escalation protocol with a total dose of 86 Gy in 43 fractions. The rate of accrual was limited by the inclusion criteria of freedom from ADT. The median follow-up for the cohort was 71 months (range 32.8-93.6 months) and the median age was 71.5 years (range 52.5-77.4 yrs). On average, 99.9% (standard deviation 0.1%) of the PTV volume received at least 77.5 Gy (V100), and 95% of the PTV volume (D95) received an average dose of 82.7 Gy (standard deviation: 1.0 Gy). The dose volume constraints were fulfilled in every patient, the mean percentage volume of rectum receiving 40 Gy, 50 Gy and 70 Gy being equal to 44.0% (±8.0), 34.9% (± 6.3) and 19.9% (± 4.7), respectively, and the mean percentage volume of bladder receiving 50 Gy and 70 Gy equal to 32.7% (±11.9) and 19.2% (± 8.2), respectively. In particular the maximum and mean dose to the rectum were 87.5 Gy (±1.2) and 42.5 Gy (±4.8), respectively; while the dose received by more than 1 and 5 cc of the rectum were 85.1 Gy (±1.3) and 79.1 Gy (±4.3), respectively.

### Toxicity

**Figure 1:**
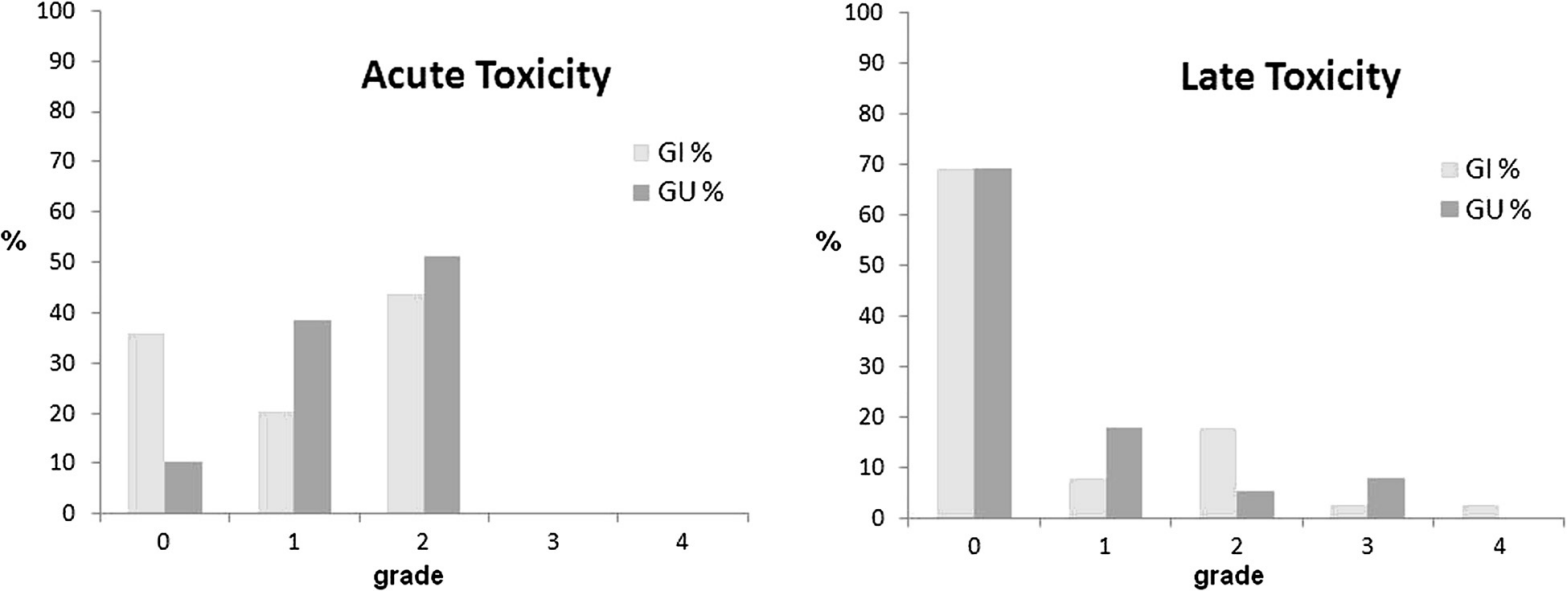
Incidence (% of patients) of acute and late gastrointestinal (GI) and genitourinary (GU) toxicity.

**Figure 2:**
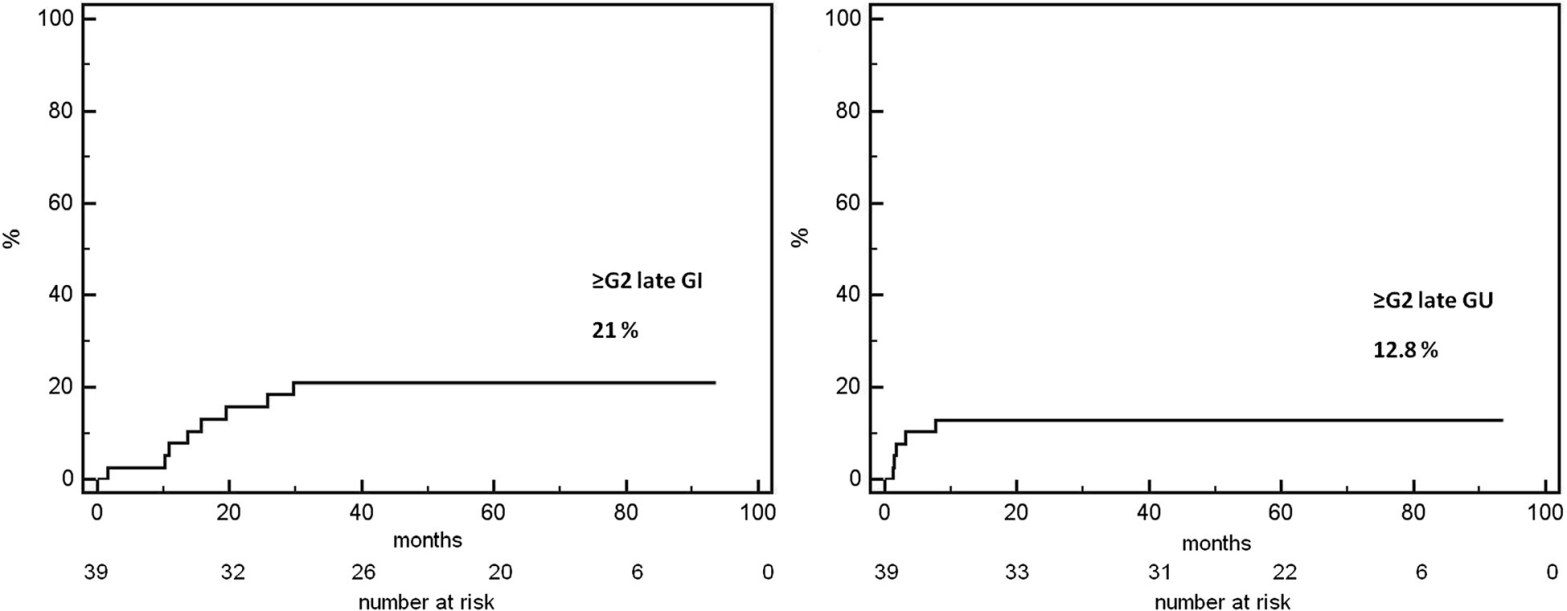
Actuarial incidence of ≥ G2 late GI and GU toxicity.

**Figure 3:**
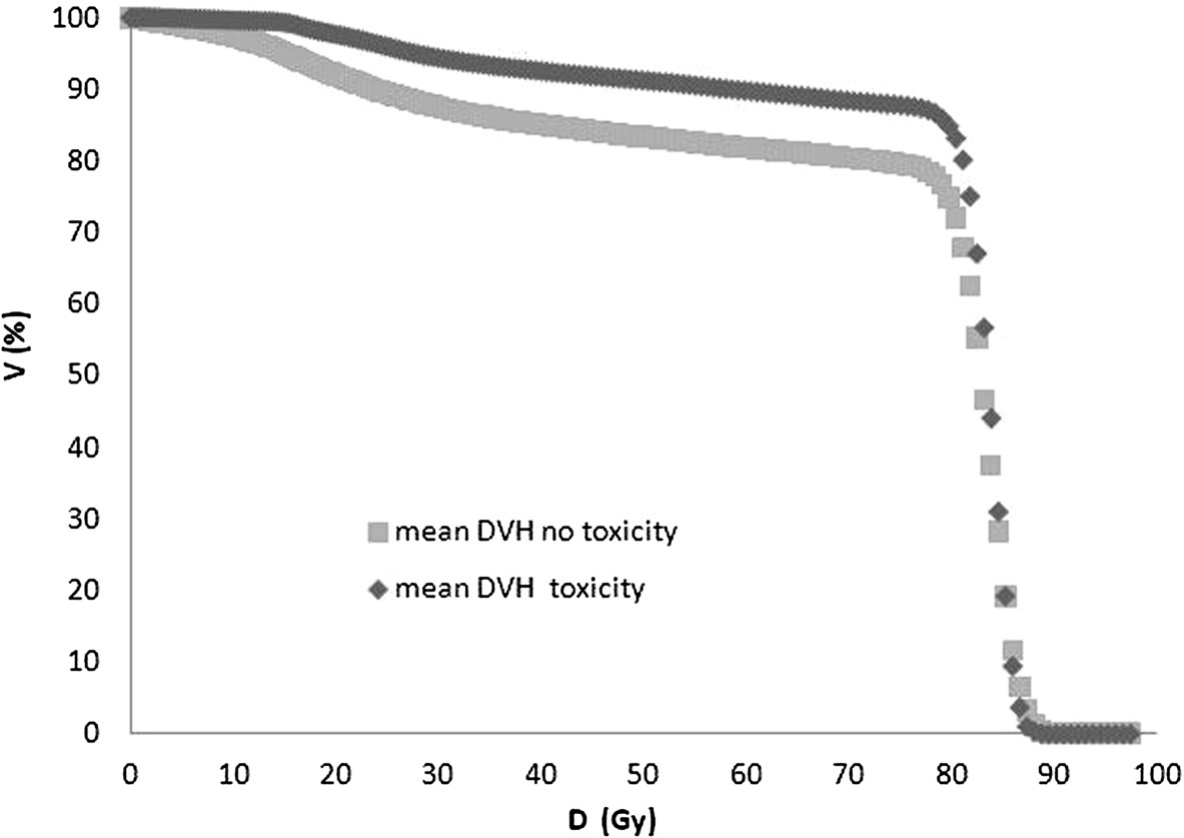
Mean dose volume histograms of the volume of rectum enclosed in the PTV for patients who did and did not experience late GI toxicity.

The IPSS questionnaire at baseline resulted in 36/39 (92%) of asymptomatic or low symptomatic patients (IPSS score ≤ 7), 3/39 (8%) moderate symptomatic (IPSS score 8–19), no patient was severely symptomatic (IPSS score 20–35). In our cohort, the acute side effects of radiotherapy were moderate and transient. No patient experienced G3 or G4 acute gastrointestinal (GI) or genitourinary (GU) toxicity. G2 acute GI and GU toxicity were observed in 17 (44%) and 20 (51%) patients, respectively (Figure 1). Fourteen patients (36%) did not experience acute GI and 4 patients (10%) did not experience acute GU toxicity. G2 late GI bleeding occurred in 7 of 39 patients (18%). Both G3 and G4 late GI toxicity were seen only in one patient (2.5%); in the first case G3 late GI toxicity was characterized by persistent bleeding treated with 4 sessions of laser coagulation, in the second case the G4 late GI toxicity was a fistula which required packing a temporary colostomy. Two patients (5%) experienced G2 late GU toxicity, while G3 late GU toxicity characterized by urethral stricture occurred in 3 patients (8%), two of whom had undergone an endoscopic transurethral resection of prostate (TURP) before radiotherapy; no patient experienced G4 late GU toxicity (Figure 1). The actuarial analysis of ≥ G2 late GI and GU complications is reported in Figure 2. The 5-year actuarial incidence of ≥ G2 late GI and GU complications was 21.0% (std error 6.6%) and 12.8% (std error 5.4%), respectively. In Figure 3 mean dose volume histograms of the volume of rectum enclosed in the PTV are shown: a statistically significant difference was found between patients who did and did not experience late ≥2 GI toxicity (p < 0.0001 Mann–Whitney test).

### Biochemical control rates and biopsies

**Figure 4:**
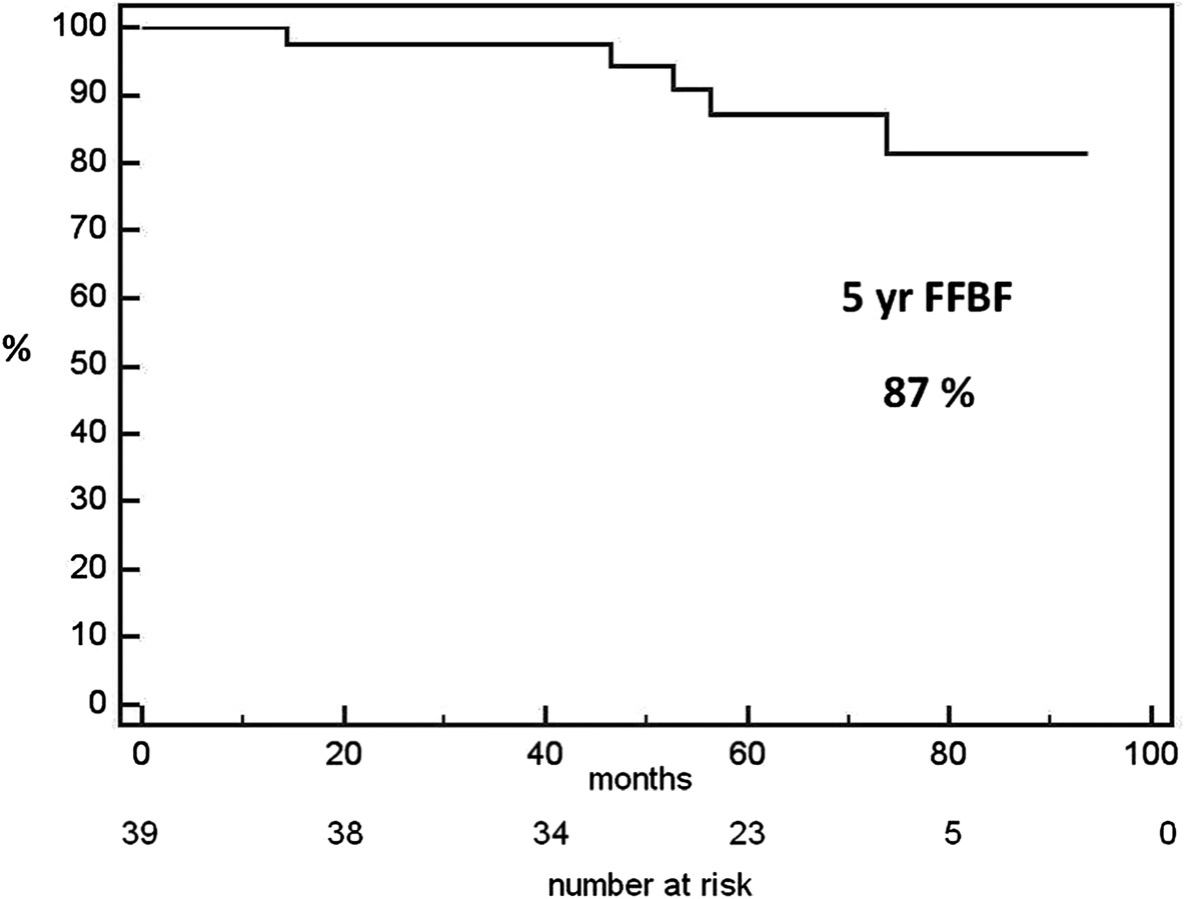
Freedom from biochemical failure survival.

The 5-year actuarial FFBF after ultra-high IMRT dose of 86 Gy at 2 Gy/fraction was 87% (standard error 6%), without the use of ADT, as shown in Figure 4. Five patients (13%) had a biochemical failure, one of these presented also a local relapse and two patients presented also distance metastasis, while 34 (87%) did not show evidence of biochemical disease. Out of 39 patients, 22 patients refused undergoing a biopsy at 2-years post-radiotherapy. Out of 17 patients who underwent re-biopsy, 15 biopsies (88%) resulted completely negative, 1 (6%) positive and 1 (6%) indeterminate, but both the last two patients did not show evidence of biochemical disease.

## Discussion

Our study represents the first prospective trial reporting results of the highest dose escalation using doses of 86 Gy at 2 Gy/fraction, for the IMRT treatment of patients with localized intermediate-risk prostate cancer without ADT. Out of 39, 7 patients (18%) reported G2 late GI toxicity, one patient (2.5%) reported G3 late GI toxicity and one patient (2.5%) reported G4 late GI toxicity. In this feasibility study, ≥G2 late GI toxicity was higher than expected from cases treated at our Institute with IMRT at doses of 80 Gy and from the literature [15-18]. However, the observed actuarial ≥ G2 late GI toxicity (21%) was lower to that found in the study RTOG 9406 conducted by Michalski et al. [29] reporting a rate of ≥ G2 GI complication ranging from 30% to 33% for 24 months at dose level V (78 Gy) but higher than that (4%) reported by Cahlon et al. [17]. The higher observed ≥ G2 late GI toxicity might be due to the lack of specific dose constraints for rectum volume within the PTV and to the fact that also seminals vesicles received the full treatment dose. In fact a statistically significant correlation was observed between dose volume histograms of the volume of rectum enclosed in the PTV and ≥ G2 late GI toxicity. It is worth noting that patients were enrolled in this study before the publication of Quantec [30], where it is stated that “Reducing the V75 by just 5% from 15% to 10% has a significant impact in the predicted complication probability …” but “the proposed dose–volume constraints might be unachievable … but every effort should be made to be as close as possible to the constraints especially in the high doses”. Nevertheless, methods allowing the reduction of the PTV, such as CBCT and/or markers for IGRT, could further reduce the incidence of rectal toxicity [31,32], considering that the prostate and the anterior rectal wall, i.e. the area most susceptible to receive an high dose, cannot be seen using EPID images only. In randomized dose-escalation trials employing 3D-CRT the incidence of ≥ G2 late GI toxicity ranged between 17% and 32% [3-7]. This GI toxicity are similar to our results, even if in our trial higher doses were delivered. Moreover, pre-radiotherapy ADT has been reported as a protective factor for GI late toxicity due to the expected reduction of PTV volume [33].

No patients experienced G4 late GU toxicity and three patients (8%) developed G3 late GU toxicity, two of which were previously treated for urethral stricture. The observed 5-year incidence of ≥ G2 late GU toxicity was 12.8%, which seems comparable to the 5-year actuarial risk (of 16%) reported by Cahlon et al. [17], and to the 3-year actuarial risk of 19% G2 late GU reported by Fonteyene et al., with doses between 72 Gy and 78 Gy [16]. However, comparisons of patients across study cohorts are difficult and should be interpreted with caution. In particular, the role of hormone therapy in the setting of dose escalation could introduce some bias, thus confounding the analysis, which needs to be evaluated in a randomized trial. The observed five years FFBF of 87%, according to the Phoenix definition, is comparable with the results of 85% reported by Cahlon et al. [17], using a total dose of 86.4 Gy (1.8 cGy/fraction) in combination with neoadjuvant or concurrent ADT. The true role of androgen deprivation in dose escalation schedules in patients with intermediate prognosis risk is currently unknown, the fact that hormonal therapy was not used in this study did not seem to impact on the outcome, even though, more patients and a longer follow up are needed to clearly state the role of ADT. Cell killing by hormone-therapy could reduce the tumor burden, enhancing local control, and maybe decreasing the rate of distant metastases [34]. Eade et al. [9] suggested that the use of doses >80 Gy for localized prostate cancer results in better local control and less distant failures when compared to doses <80 Gy, analyzing a cohort of patients free from ADT. In this report, the authors observed a reduced risk of biochemical recurrence of 2.2% at 8 years for the addition of each Gy over 80 Gy and concluded that the plateau on the dose–response curve for prostate cancer lies well above 80 Gy. Also, feasibility studies of single Institutions and some randomized trials of dose escalation showed improved results in the treatment of localized prostate cancer [1-8]; analyzing the effects of increased doses between prognostic categories, the best results are observed in the intermediate risk [3-9,15,34-36]. Even though, with a larger number of enrolled patients a multivariate analysis could better clarify the results observed, we believe that the current series demonstrates the advantage in terms of disease control of using ultra-high doses in the treatment of intermediate risk prostate cancer while the incidence of toxicity observed could be lowered by applying stricter requirements on the dose volume constraints at the interface of the rectum with the posterior portion of the prostate gland and introducing a more advanced imaging protocol, i.e. cone beam CT imaging. Moreover, authors are aware that quality of life questionnaires to investigate treatment effects as reported by patients could have added information to the overall rating of treatment results; for this reason, since then, great effort has been made to introduce in our policy also this additional tool of evaluation.

## Conclusion

Our results proved to be good in terms of FFBF without using ADT in intermediate-risk prostate cancer patients. Although a longer follow up and more patients are needed to assert that ultra-high dose escalation using IMRT could be a viable alternative to lower doses plus ADT, though manifesting an increase of ≥ G2 late GI rate, the data reported in this study seem promising. Finally, even if the inclusion criteria of freedom from ADT is a very limiting factor for the accrual rate in the intermediate risk patient cohort because ADT is often a standard therapeutic strategy, we believe that only a randomized study can accurately compare outcomes between different doses in dose escalation schedules.

## Abbreviations

GI: Gastrointestinal; GU: Genitourinary; FFBF: Freedom from biochemical failure; IMRT: Intensity modulated radiation therapy; ADT: Androgen deprivation therapy; DMFS: Distant metastasis-free survival; CSS: Cancer-specific survival; 3D-CRT: Three-dimensional conformal radiation therapy; CTV: Clinical target; PTV: Planning target volume; EPID: Electronic portal imaging device; DRR: Digitally reconstructed radiography; DRE: Rectal examination; TURP: Transurethral resection of prostate.

## Competing interests

The authors hereby declare that they do not have any competing interest in this study.

## Authors’ contribution

MGP, GA, VL and BS conceived and designed the study. MGP, VL, BS, SG, SA, GI, PP collected and assembled the data, VL performed the statistical analysis, MGP and VL wrote the manuscript. LS and GA gave support in the final drafting of the paper. All authors read and approved the final manuscript.
